# Genomic expression program of *Saccharomyces cerevisiae* along a mixed-culture wine fermentation with *Hanseniaspora guilliermondii*

**DOI:** 10.1186/s12934-015-0318-1

**Published:** 2015-08-28

**Authors:** Catarina Barbosa, Arlete Mendes-Faia, Patrícia Lage, Nuno P. Mira, Ana Mendes-Ferreira

**Affiliations:** Escola de Ciências da Vida e Ambiente, Universidade de Trás-os-Montes e Alto Douro, Vila Real, Portugal; BioISI-Biosystems and Integrative Sciences Institute, Campo Grande, Lisbon, Portugal; iBB-Institute for Bioengineering and Biosciences, Avenida Rovisco Pais, 1049-001 Lisbon, Portugal; Department of Bioengineering, Instituto Superior Técnico, Avenida Rovisco Pais, 1049-001 Lisbon, Portugal

**Keywords:** Mixed-culture fermentation, Transcriptomics, *Saccharomyces cerevisiae*, *Hanseniaspora guilliermondii*, Wine

## Abstract

**Background:**

The introduction of yeast starter cultures consisting in a blend of *Saccharomyces cerevisiae* and non-*Saccharomyces* yeast strains is emerging for production of wines with improved complexity of flavor. The rational use of this approach is, however, dependent on knowing the impact that co-inoculation has in the physiology of *S. cerevisiae*. In this work the transcriptome of *S.cerevisiae* was monitored throughout a wine fermentation, carried out in single culture or in a consortium with *Hanseniasporaguilliermondii*, this being the first time that this relevant yeast–yeast interaction is examined at a genomic scale.

**Results:**

Co-inoculation with *H. guilliermondii* reduced the overall genome-wide transcriptional response of *S. cerevisiae* throughout the fermentation, which was attributable to a lower fermentative activity of *S. cerevisiae* while in the mixed-fermentation. Approximately 350 genes *S. cerevisiae* genes were found to be differently expressed (FDR < 0.05) in response to the presence of *H. guilliermondii* in the fermentation medium. Genes involved in biosynthesis of vitamins were enriched among those up-regulated in the mixed-culture fermentation, while genes related with the uptake and biosynthesis of amino acids were enriched among those more expressed in the single-culture. The differences in the aromatic profiles of wines obtained in the single and in the mixed-fermentations correlated with the differential expression of *S. cerevisiae* genes encoding enzymes required for formation of aroma compounds.

**Conclusions:**

By integrating results obtained in the transcriptomic analysis performed with physiological data our study provided, for the first time, an integrated view into the adaptive responses of *S. cerevisiae* to the challenging environment of mixed culture fermentation. The availability of nutrients, in particular, of nitrogen and vitamins, stands out as a factor that may determine population dynamics, fermentative activity and by-product formation.

**Electronic supplementary material:**

The online version of this article (doi:10.1186/s12934-015-0318-1) contains supplementary material, which is available to authorized users.

## Background

Various non-*Saccharomyces* yeasts have been examined as potential adjuncts to *Saccharomyces cerevisiae* exploiting their flavor properties in order to respond to the new challenges of consumer demands for wines with high complexity of flavor and stylistic distinction [1–5]. This beneficial impact of non-*Saccharomyces* yeasts on wine composition has been found to be influenced by the species/strains of *Saccharomyces* and non-*Saccharomyces* used; by the size of the inocula and by the timing of inoculation (simultaneous *vs* sequential), among other factors [reviewed in 2]. On the other hand, non-*Saccharomyces* yeasts have also been found to have an inhibitory effect over *S. cerevisiae* growth, presumably due to the production of toxic compounds such as fatty acids and killer factor [6–10]. In addition, competition for nutrients, in particular nitrogen and/or vitamins, were also proposed to limit growth and fermentative ability of *S. cerevisiae* strains when co-cultured with non-*Saccharomyces* species [7, 11, 12]. Moreover, a recent study from our laboratory has shown that initial nitrogen levels of musts impact mixed-culture dynamics and final aroma composition of wines [6].

Wine research has benefited enormously from the privileged position of *S. cerevisiae* as an experimental system in life sciences research [13]. The budding yeast was the first eukaryote organism to have its genome sequenced [14], which paved the way for the development of robust advanced genetic tools that put this species at the forefront of ‘-omics’ research. Using these genome-wide approaches, previous studies have elucidated cellular adaptive responses of *S. cerevisiae* during wine fermentation at different genomic levels including transcriptome, proteome and metabolome [15–22]. In particular, transcriptomic analysis has provided valuable insights to understand the molecular basis by which the nutritional composition of the growth medium and, in particular the initial concentration of nitrogen, impacts growth and performance of fermentations undertaken by *S*. *cerevisiae* wine yeasts [15, 19, 23, 24]. This information, besides giving basic knowledge on *S. cerevisiae* physiology, has provided valuable data of practical interest for the control and prevention of slow and premature fermentation arrest during winemaking and for the clarification of the impact of nitrogen metabolism of *S. cerevisiae* on aroma compounds formation during alcoholic fermentations. OMICS analyses also have the potential to provide a clear cut picture of the molecular mechanisms by which *S. cerevisiae* responds to the presence of other microbes in the environment, however, up to now only a few studies have addressed that issue in the context of wine fermentations. Recently, the transcriptome-wide response of yeast cells in mixed cultures with different wine bacteria had been elucidated including *Lactobacillus delbrueckii subsp. bulgaricus*, which co-occur with yeast in kefir fermentations [25] and *Oenococcus oeni*, used for malolactic fermentation [26–28, 29]. The results of this last study indicate that *S. cerevisiae*-*O. oeni* interaction during winemaking involves not only indirect competition for nutrients, but also direct antagonistic responses. Although yeast–yeast interactions have not been examined at a genome-wide scale, some attempts have been made to examine the influence exerted by the presence of *Starmerella bombicola*, on the expression of a few selected *S. cerevisiae* genes [30]. Exposure to the non-*Saccharomyces* species was found to lead to alterations in both expression and enzymatic activity of *S. cerevisiae* alcohol dehydrogenase 1 (encoded by ADH1 gene) and pyruvate decarboxylase (encoded by PDC1).

In this study it was performed the first genome-wide analysis of how *S*. *cerevisiae* adjusts its transcriptome along fermentation of a natural grape must in single culture or in consortium with *Hanseniasporaguilliermondii*. To our knowledge this is the first study focused on the elucidation at the molecular level of this yeast–yeast interaction, a knowledge that could be used to guide the rational development of mixed blends composed by these two yeasts and of its subsequent utilization in mixed fermentations.

## Results

In a previous work the effect of grape-juice nitrogen availability on wine yeast mixed-culture fermentations has been evaluated using a strain of *H. guilliermondii* in consortium with *S. cerevisiae* [6]. The results obtained provided evidences that the presence of *H. guilliermondii* negatively affects *S. cerevisiae* growth and fermentation rate, irrespective of the initial nitrogen concentration of the grape-juice. Co-inoculation of *S. cerevisiae* with *H. guilliermondii* has also been found to significantly alter the panoply of aroma compounds found at the end of the fermentation [6]. In this work the alterations occurring in the transcriptome of *S. cerevisiae* along a mixed wine-fermentation with *H. guilliermondii* were monitored using DNA microarrays. Since *H. guilliermondii* is a non-standard model yeast for which comprehensive DNA microarrays are not available, we have focused on the effect of the co-inoculation only in the alteration of the *S. cerevisiae* transcriptome. The experimental conditions used were the same as those described in [6], being of notice the choice of cultivating the two yeasts in natural grape-juice supplemented with di-ammonium phosphate (DAP), as these were the conditions where the impact of co-inoculation on the formation of aroma compounds was more evident [6].

### Transcriptional profiling of *S. cerevisiae* in single- and in mixed-culture fermentations

**Fig. 1:**
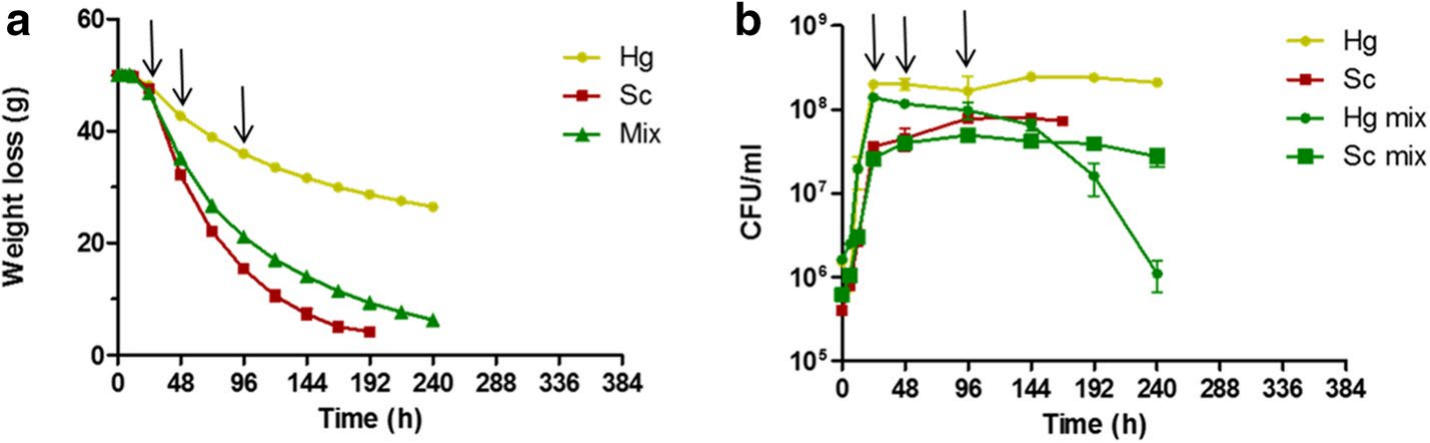
Fermentation kinetics (**a**) and growth profiles (**b**) of single- or mixed-cultures of *S. cerevisiae* and *H. guilliermondii* in natural grape-juice. Values presented are the means from triplicate fermentations. *Arrows* indicate the sampling points for transcriptomic analysis (The data stem from Lage et al. [6])

**Fig. 2:**
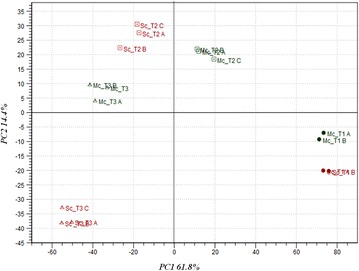
Principal Component Analysis of the alterations registered in the transcriptome of *S. cerevisiae* along a wine fermentation performed in single culture or in consortium with *H. guilliermondii.* The PCA plot shows variation in expression levels of S. *cerevisiae* genes either in single- (Sc) or mixed-culture (Mc) at each fermentation stage (24, 48 and 96 h)

**Fig. 3:**
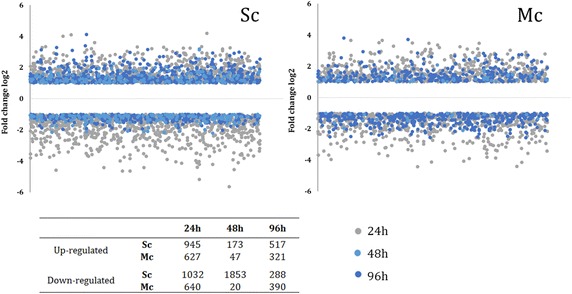
Variation of the expression of *S. cerevisiae* genes in single or in mixed culture with *H. guilliermondii*. The expression of each *S. cerevisiae* gene after 24, 48 or 96 h of single or mixed wine fermentation was compared with its mean expression value along the fermentation. Genes exhibiting at least twofold difference in expression were considered to be differently expressed and were included in this analysis

The transcriptomic profiling of the mixed-culture fermentations was performed at three different time-points (Fig. 1; Table 1): in mid-exponential growth phase (24 h), in early stationary-phase (48 h), and in late stationary growth-phase (96 h). To get a global view on how the presence of *H. guilliermondii* impacted the transcriptome of *S. cerevisiae* throughout the fermentation, the data obtained from the microarrays experiments were subjected to Principal Component Analysis (PCA). This multivariate statistical analysis revealed that gene expression differences between the fermentation stages were much greater than those observed between the two inoculum types (Fig. 2). The first two principal components (PCs) accounted for more than 75 % of the variation observed, with PC1 accounting for the majority (61.8 %) of the observed variability. Samples clustered together in a fermentation stage-specific manner, grouping along the first axes of variation, being observed minor variations between the independent biological replicates. Nevertheless, the separation of the samples collected at the same time-point rendered clear that the presence of *H. guilliermondii* affected *S. cerevisiae* transcriptome along fermentation. Notably, the maximal variation in *S. cerevisiae* genomic expression was reached at the later fermentation stages, in agreement with the much higher number of genes that was found to be differentially in the pair-wise comparisons performed between the two fermentations at the same time-point (see below, Additional file 1). As denoted by Maligoy et al. [27] caution should be taken when analyzing transcriptome data from two parallel cultures, since the variations of transcript levels observed could be either specific to the comparison of the two culture conditions or linked to a difference in the dynamics of the two cultures. To assure that the observed changes in the expression of *S. cerevisiae* genes truly reflects the influence of the presence of *H. guilliermondii*, rather than being attributable to different fermentation stages of the mixed and single cultures, the expression of a given gene in a given fermentation stage was compared to its mean expression (calculated taking the average of the expression levels obtained in the three time points analyzed). Although the mean expression value of each gene along the fermentation is merely an arbitrary reference point, such way of analyzing gene expression mitigates the influence exerted by fermentation dynamics, while maintaining the aptitude to identify expression differences [31]. Furthermore, this approach also has the advantage of providing information on how *S. cerevisiae* transcriptome adjusts to the different dynamics of the single or mixed-culture fermentation; an information that would be missed if only cross-comparisons between expression levels in single vs mixed cultures had been performed. Only genes having an increased or decreased expression of at least twofold were considered to be up- or down- regulated in a given fermentation stage. Using this criterion, two sets of 2224 genes and 1406 *S. cerevisiae* genes were considered to be differently expressed along the single- or mixed-fermentations, respectively (Additional files 2, 3). K-mean clustering analysis of these genes revealed that the modifications of *S. cerevisiae* genomic expression occurring throughout the wine fermentations showed similar patterns in the single and in the mixed culture since the gene clusters obtained for the two datasets are, in general, the same (Additional files 2, 3). A closer look into the functional categories of genes included in each cluster revealed that the herein observed alterations of the *S*. *cerevisiae* transcriptome along wine fermentation, either in single or in mixed-culture, are consistent with the results reported in other studies carried out with different *S. cerevisiae* strains and/or exploring different fermentation conditions [17, 19–21, 32]. In specific, genes involved in carbohydrate metabolism, mitochondrial respiration/oxidative phosphorylation, stress response were found to be induced at 48 h of fermentation, both in the single- (clusters II–IV; Additional file 2) and in the mixed-culture fermentation (clusters I–III and IX; Additional file 3), this being attributed to the higher fermentative activity exhibited by the yeast cells at this fermentation stage. Differently, genes involved in cell growth, protein biosynthesis and ribosomal processing, were found to have higher expression at the earlier fermentation stage being repressed afterwards in response to stress associated with alcoholic fermentation progression and entrance in stationary phase. The fact that *S. cerevisiae* in single-culture displayed more noticeable changes in its transcriptome, in terms of both the number of genes and the magnitude of expression changes, compared to mixed culture (Fig. 3), might reflect a higher need to adjust to a more challenging environment caused by the higher fermentative activity observed.

**Table 1:** Overview of some fermentation parameters determined at the time-points selected for transcriptomic analysis

Sampling point	Glucose (g/L)	Fructose (g/L)	Ethanol (% v/v)	Ammonium (mg/L)	Glycerol (g/L)
24 h					
Sc	104.31 ± 10.4^a^	110.89 ± 6.47^ab^	1.97 ± 0.07^d^	167.96 ± 19.24^a^	1.00 ± 0.13^e^
Mc	109.31 ± 5.86^a^	113.21 ± 5.29^a^	1.74 ± 0.10^d^	171.69 ± 21.15^a^	1.64 ± 0.14^d^
48 h					
Sc	57.29 ± 5.77^c^	93.18 ± 3.41^b^	4.80 ± 0.28^c^	1.91 ± 1.10^c^	4.74 ± 0.45^c^
Mc	76.25 ± 2.72^b^	93.44 ± 1.91^b^	5.07 ± 0.54^c^	39.10 ± 8.46^b^	4.81 ± 0.49^c^
96 h					
Sc	4.98 ± 1.64^e^	52.44 ± 1.32^c^	8.48 ± 0.07^b^	nd	6.89 ± 0.19^b^
Mc	28.28 ± 5.09^d^	55.25 ± 5.58^c^	9.58 ± 0.43^a^	nd	8.51 ± 0.35^a^

Data points are the means from triplicate fermentations

*Sc* single-culture, *Mc* mixed-culture, *nd* not detected

Values in the same column with different superscript letters are significantly different (p < 0.05)

### Inference of the dynamics of transcriptional regulatory networks underlying the control of *S. cerevisiae* transcriptome throughout single and mixed fermentations

**Fig. 4:**
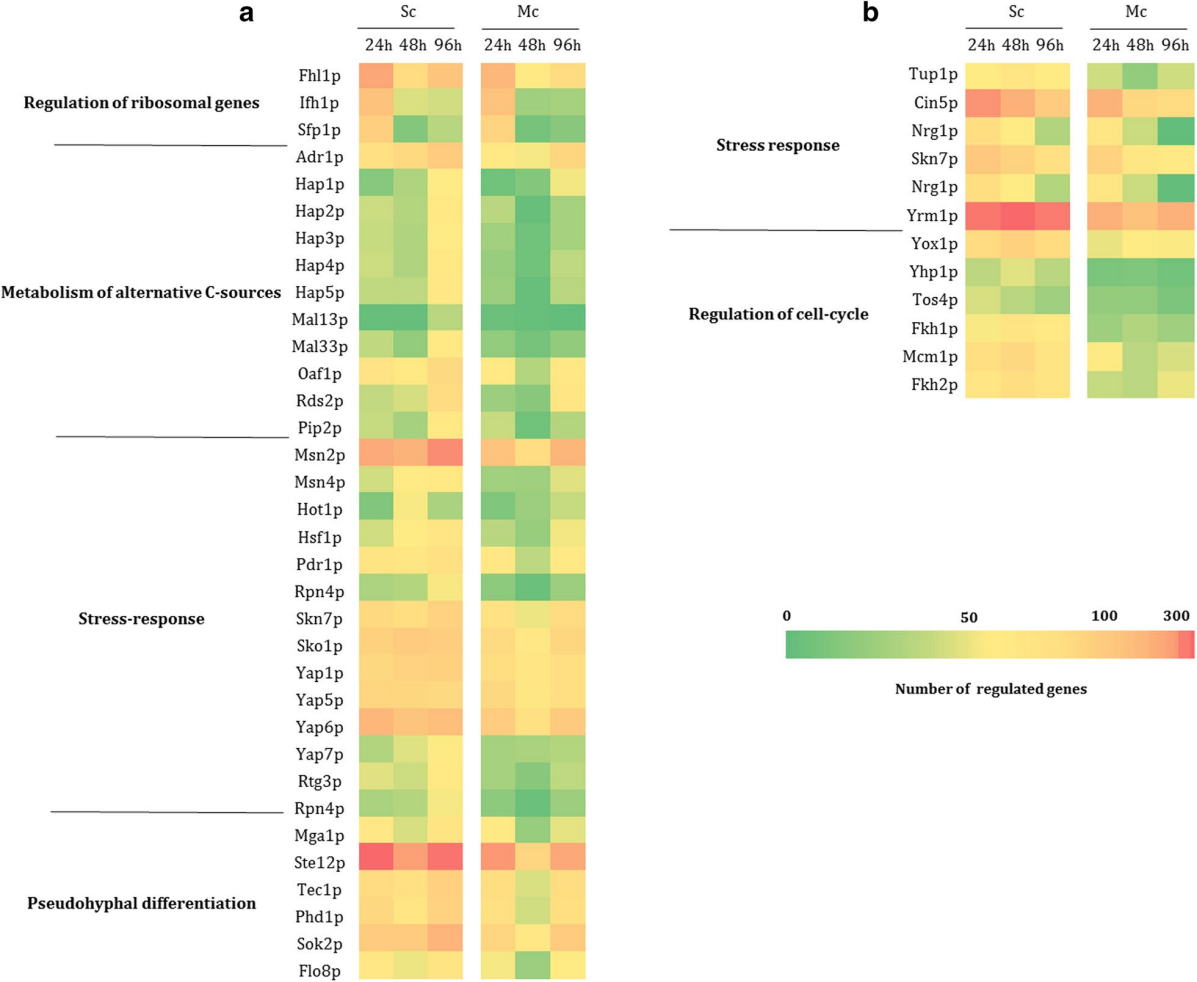
Association between *S. cerevisiae* genes whose expression changed along the single or mixed wine fermentations with their documented regulators. The entire dataset of genes found to change their expression throughout the single or the wine fermentations was searched for documented targets of all described *S. cerevisiae* transcription factors using the tools and information available in the YEASTRACT database. The activity of each transcription factor was predicted based on the number of targets present in each dataset only considering direct regulatory associations in which binding of the transcription factor to the target gene promoter. The dataset of up-regulated genes was only searched for targets of transcriptional activators (**a**) while the dataset of down-regulated genes was only searched for targets of transcriptional repressors (**b**). Transcriptional regulators found to work both as transcriptional activators or repressors were included in both analyses. In this figure only a selected set of regulatory associations is shown but the full list is available in Additional file 1

The expression and the activity of transcriptional regulators have been shown to be on the basis of different metabolic/phenotypic traits of fermentations undertaken by different wine yeast strains [33]. In that sense, to better understand how co-inoculation with *H. guilliermondii* affected the overall *S. cerevisiae* regulatory network along the fermentation, the datasets of the differently expressed genes in the three time-points herein under study were analyzed using the tools available in the YEASTRACT database [34, 35]. The activity of each transcription factor along the two fermentations in each of the time points was predicted based on the number of documented targets in the corresponding datasets considering only direct regulatory associations. The results obtained were compiled in heat maps, which are shown in Fig. 4 and in Additional file 1. An over-representation of genes regulated by Sfp1, Fhl1 and Ifh1 is observed in the dataset of genes up-regulated after 24 h in both single and mixed-culture fermentations (Fig. 4a). These transcription factors are involved in regulation of ribosomal gene expression and their pattern of activity is consistent with the early up-regulation of these genes during the growth phase and subsequent repression once cells approach stationary phase, as discussed above. Within the dataset of genes up-regulated after 48 and 96 h in the single culture fermentation it is clear the enrichment of documented targets of the Adr1, Hcm1, Hap1, Hap2, Oaf1 and Pip2 transcription factors, all positive regulators of genes required for the use of alternative carbon sources (Fig. 4a). The activation of these transcription factors at these stages of the fermentation could be attributable to an alleviation of glucose repression which has been suggested to occur along wine fermentations, a response that was proposed to be mediated by Adr1, Cat8 and the members of the Hap complex [19, 20]. Notably, the relevance of the above-referred regulons was much less prominent in the dataset of genes up-regulated in the mixed-culture fermentation (Fig. 4b), which could be due to the much lower consumption of glucose that was registered in this fermentation, compared to the single-culture fermentation (Table 1). Over-representation of the regulons controlled by several stress-responsive transcription factors, including Msn2 and Msn4, already demonstrated to play an important role in the control of transcriptional response to “fermentation stress” [15–22], was also evident in the three fermentation points analyzed, more pronounced at 48 and 96 h (Fig. 4). In the mixed-culture fermentation, the over-representation of these “stress-responsive” regulons was considerably less prominent, suggesting that the environment of the mixed fermentation could be less stressful for *S. cerevisiae* cells than the environment of the single-culture fermentation, as discussed above. Significantly, Sko1, Hot1 and Skn7, three of the stress-responsive factors that emerged from our analysis, are all known to be become activated upon phosphorylation by the Hog1 kinase [36], which was found to play an essential role in *S. cerevisiae* ability to ferment grape-juice medium [37]. Several positive regulators of pseudohyphal growth were also found to be over-represented in the dataset of genes up-regulated throughout the single and mixed-culture fermentations, albeit in this last dataset the enrichment is less pronounced (Fig. 4b). Previous studies have also reported different levels of expression and activity of Phd1 and Sok2 in different wine yeast strains and in this case Sok2 activity was correlated with the different metabolic properties of the strains analyzed [33]. It is of notice the fact that the enrichment of these regulons related with pseudohyphal differentiation was less significant in the mixed culture fermentation (Fig. 4b). This difference could be attributable to the lower consumption of ammonium, considering the essential role played by nitrogen availability in the control of transition to pseudohyphal differentiation [38].

Within the dataset of down-regulated genes it is evident an over-representation of genes regulated by the stress-responsive transcription factors Skn7, Yap6 and Cin5. All these transcription factors had been found to recruit the general transcriptional repressor Tup1, a response that is thought to contribute to fine-tune the balance between activated and repressed genes in response to changing environment [39]. A similar function has also been attributed to Nrg1 [40], another transcription factor found to be over-represented in the dataset of genes repressed throughout the two fermentations (Fig. 4). Interestingly, a significantly high number of documented targets of the drug-responsive transcription factor Yrm1 was found in the dataset of genes down-regulated along the single and mixed-culture fermentations (Fig. 4). Until so far the role of Yrm1 in wine fermentation has not been examined although previous transcriptomic analysis have suggested that transcription factors involved in the control of pleiotropic drug response may play a role in the control of *S. cerevisiae* genomic expression along wine fermentations [20, 41].

### Co-inoculation with *H. guilliermondii* elicits dissimilar transcriptional responses in *S. cerevisiae*

In this section the expression of *S. cerevisiae* genes in mixed culture and in single culture is compared to have a clearer picture of the effect exerted by the presence of *H. guilliermondii* in the growth medium. Since the dynamics of the two fermentations were not significantly different, as discussed above, the differences found in gene expression in the two culture conditions are likely to result from *S. cerevisiae* response to the presence of *H. guilliermondii*. To identify genes that could discriminate the two inoculation strategies used, a Rank-Product (RP) analysis was performed considering all samples of the single-culture fermentation as a group and those of mixed-culture fermentation as another group, irrespective of the fermentation stage. This unsupervised approach led to the identification of 120 *S. cerevisiae* genes that seem to respond to the presence of *H. guilliermondii* during the course of fermentation, 85 being up-regulated in the presence of the non-*Saccharomyces* species and 35 down-regulated. A list of the top 10 *S. cerevisiae* genes whose expression varied the most in the presence of *H. guilliermondii* is shown in Table 2. In general, the majority of the differently expressed genes are involved in amino acid biosynthesis, uptake or catabolism of specific amino acids for nitrogen mobilization, biosynthesis of vitamins, and purine nucleotide biosynthetic process, as well as an important number of genes with no biological function associated (Table 3; Additional file 4). In the following section, the results obtained at each time point are separately discussed.

**Table 2:** Top10 of the genes differently expressed in *S. cerevisiae* in single-culture (Sc) and mixed-culture (Mc) fermentations, at the three fermentation stages (24, 48 and 96 h)

ORF	Gene	Function	Fold change Sc/Mc
24 h			
YCL025C	*AGP1*	Low-affinity amino acid permease with broad substrate range	13.4
YDR508C	*GNP1*	High-affinity glutamine permease	8.1
YOL086 W-A		Molecular function unknown	5.7
YHR021 W-A	*ECM12*	Putative protein of unknown function	5.3
YKL183C-A		Putative protein of unknown function	5.1
YOR348C	*PUT4*	Proline permease	4.8
YDR130C	*FIN1*	Spindle pole body-related intermediate filament protein	4.6
YBL042C	*FUI1*	High affinity uridine permease	4.5
YAL037C-A		Putative protein of unknown function	4.4
YBL052C	*SAS3*	Histone acetyltransferase activity	4.0
YHR044C	*DOG1*	2-deoxyglucose-6-phosphate phosphatase	−11.8
YDR018C		Transferase activity, transferring acyl groups	−8.9
YPL258C	*THI21*	Hydroxymethylpyrimidine (HMP) and HMP-phosphate kinase; involved in thiamine biosynthesis	−7.9
YDL021 W	*GPM2*	Molecular function unknown	−7.1
YHR043C	*DOG2*	2-deoxyglucose-6-phosphate phosphatase	−6.7
YCR020C	*PET18*	Protein of unknown function	−6.1
YLR176C	*RFX1*	Major transcriptional repressor of DNA-damage-regulated genes	−6.1
YHL048C-A		Putative protein of unknown function	−5.9
YOL055C	*THI20*	Trifunctional enzyme of thiamine biosynthesis, degradation and salvage	−5.6
YHR076 W	*PTC7*	Type 2C serine/threonine protein phosphatase (PP2C)	−5.5
48 h			
YLR142 W	*PUT1*	Proline oxidase involved in utilization of proline as sole nitrogen source	55.3
YJR152 W	*DAL5*	Allantoate permease	52.7
YKR039 W	*GAP1*	General amino acid permease	25.3
YMR107 W	*SPG4*	Molecular function unknown	23.3
YMR175 W	*SIP18*	Phospholipid binding	21.2
YMR118C		Putative mitochondrial inner membrane protein of unknown function	20.3
YPR194C	*OPT2*	Oligopeptide transporter	18.3
YCR098C	*GIT1*	Plasma membrane permease; mediates uptake of glycerophosphoinositol and glycerophosphocholine as sources of the nutrients inositol and phosphate	17.4
YHL016C	*DUR3*	Plasma membrane transporter for both urea and polyamines	15.4
YCL064C	*CHA1*	Catabolic l-serine (l-threonine) deaminase	13.8
YMR095C	*SNO1*	Protein of unconfirmed function; involved in pyridoxine metabolism; expression is induced during stationary phase	−14.2
YCL026C-A	*FRM2*	Type II nitroreductase, using NADH as reductant	−13.7
YGL117 W		Putative protein of unknown function	−12.9
YBR092C	*PHO3*	Acid phosphatase activity	−10.1
YMR094 W	*CTF13*	Subunit of the CBF3 complex	−9.9
YML116 W	*ATR1*	Multidrug efflux pump of the major facilitator superfamily	−8.2
YML123C	*PHO84*	High-affinity inorganic phosphate (Pi) transporter	−8.0
YLR372 W	*SUR4*	Elongase; involved in fatty acid and sphingolipid biosynthesis	−7.8
YGL162 W	*SUT1*	Transcription factor of the Zn(II)2Cys6 family; positively regulates genes involved in sterol uptake under anaerobic conditions	−7.2
YBR249C	*ARO4*	3-deoxy-D-arabino-heptulosonate-7-phosphate (DAHP) synthase	−6.9
96 h			
YEL061C	*CIN8*	Kinesin motor protein	86.2
YJL051 W	*IRC8*	Bud tip localized protein of unknown function	43.0
YJL148 W	*RPA34*	RNA polymerase I subunit A34.5	28.6
YNL129 W	*NRK1*	Nicotinamide riboside kinase	27.6
YLR265C	*NEJ1*	Protein involved in regulation of non homologous end joining	25.9
YOR177C	*MPC54*	Component of the meiotic outer plaque	21.9
YOR305 W	*RRG7*	Protein of unknown function	21.0
YLR151C	*PCD1*	8-oxo-dGTP diphosphatase	21.0
YKL011C	*CCE1*	Mitochondrial cruciform cutting endonuclease	21.0
YDR523C	*SPS1*	Putative protein serine/threonine kinase	19.5
YBR194 W	*AIM4*	Protein proposed to be associated with the nuclear pore complex	−4.5
YOR090C	*PTC5*	Mitochondrial type 2C protein phosphatase (PP2C)	−3.6
YGR213C	*RTA1*	Protein involved in 7-aminocholesterol resistance	−3.3
YDR434 W	*GPI17*	Transmembrane protein	−3.2
YBR111C	*YSA1*	Nudix hydrolase family member with ADP-ribose pyrophosphatase activity	−3.2
YOL131 W		Putative protein of unknown function	−3.2
YER061C	*CEM1*	Mitochondrial beta-keto-acyl synthase	−3.1
YNR058 W	*BIO3*	7,8-diamino-pelargonic acid aminotransferase (DAPA)	−3.1
YCL032 W	*STE50*	Adaptor protein for various signaling pathways	−3.0
YOR353C	*SOG2*	Key component of the RAM signaling network	−3.0

**Table 3:** Distribution in functional categories of the genes significantly (FDR < 0.05) higher expressed in *S. cerevisiae* in mixed-culture (Mc) and in single-culture (Sc) fermentations, irrespective of the fermentation stage

k	f	p-value	Category
Sc			
3	7	6.94E−05	Allantoin catabolic process
2	2	1.64E−04	Urea catabolic process
11	815	1.62E−03	Transmembrane transport
Mc			
3	17	8.82E−06	Biotin biosynthetic process
6	110	2.04E−05	Biosynthesis of vitamins, cofactors, and prosthetic groups
5	98	1.48E−04	Cellular amino acid biosynthetic process
3	29	4.54E−04	Purine nucleotide/nucleoside/nucleobase anabolism

k represents the number of genes of each category that appears in our experiment. f is the total number of genes in that category and p-value (single hypothesis one-sided P value of the association between the total number of genes and the genes that are differentially expressed)

#### Fermentation stage 1 (24 h)

In the pair wise comparison performed at 24 h only 27 genes were found to be differentially expressed (FDR < 0.05) between the single and mixed-culture fermentations (Additional file 4). Interestingly among the ten genes that were more expressed in *S. cerevisiae* in the single-culture, compared to the mixed culture, was *GAP1* (8.7-fold) and *AGP1* (13.4-fold), encoding general amino acid carriers with broad substrate ranges, as well as *PUT4* (4.8-fold), encoding a specific proline transporter. These three genes are under the nitrogen catabolite repression (NCR) and their higher expression in the single-culture might suggest an alleviation of this repressive effective. Consistent with this idea, the NCR-repressed *MEP1* and *MEP2* genes, encoding the specific permeases for ammonium assimilation, were also found to be more actively transcribed in the single-culture fermentation than in the mixed fermentation, 1.97- and 1.95- fold, respectively. Although amino acid consumption profile was not assessed in this study, high levels of ammonium were detected at 24 h in both fermentations (Table 1). In this context, our results suggest that the presence of *H. guilliermondii* could be restraining the efficient assimilation of nitrogen compounds available in grape-juice by *S. cerevisiae*, this being in line with the results of a previous report [42]. Thus, the higher expression of these genes involved in the uptake and utilization of alternative nitrogen sources in single-culture fermentation may reflect a higher yeast cells ability to scavenge for nitrogen available in fermentation medium. On another hand 17 *S. cerevisiae* genes were found to have an increased expression in the mixed-culture (Additional file 4). Among them we found *THI20* (5.6-fold), and *THI21* (7.9-fold), whose expression is regulated in the dependence of thiamine availability [43]. Thiamine has a pivotal role in fermentative activity as it is necessary for the biosynthesis of thiamine-pyrophosphate, a cofactor essential for the activity of pyruvate decarboxylase. The higher expression of *THI20* and *THI21* in the mixed-culture suggest that *S*. *cerevisiae* and *H. guilliermondii* might be competing for thiamine which could lead to a depletion of this vitamin in the must.

Indeed, depletion of thiamine in musts in co-cultures of *S*. *cerevisiae* with *Kloeckera apiculata* have been reported leading to a reduction in the fermentation rate and to higher levels of glycerol in final wines [7]. Notably, our results are in line with these observations, as in addition to the lower fermentative activity noticed in mixed-culture fermentations, a higher amount of glycerol was produced in the mixed-fermentation (Table 1). Similar results were obtained by Milanovic et al. [30] while studying wine mixed-culture fermentations with *Starmerella bombicola* and *S. cerevisiae*. As also seen herein, mixed-culture produced more glycerol and faster than *S. cerevisiae* single culture. From the winemaking point of view, these effect exerted by non-*Saccharomyces* species, including *H. guilliermondii,* are very interesting as, although it has no direct impact on the aromatic characteristics, wines can benefit from an increased glycerol production to improve the mouth feel and perceived sweetness of wine.

#### Fermentation stage 2 (48 h)

At 48 h we found *S. cerevisiae* 186 genes differentially expressed between the single and the mixed fermentations, 77 being more expressed in the single-culture fermentation, and 109 genes more expressed in the mixed-culture fermentation (Additional file 4) (Table 2). Among the set of genes found to be more expressed in the single culture we found 20 genes (*p* value 1.9 × 10^−12^) included in the so-called Fermentation Stress Response (FSR) [20]. The increased expression of these genes in the single-culture could be correlated with a higher activity of *S. cerevisiae* in single-culture, as discussed above. As observed after 24 h of fermentation, several genes more expressed in single-culture are known to be under the NCR response, namely those encoding proteins required for the uptake and utilization of allantoin—*DAL5* (52.7-fold more expressed in the single-culture), *DAL4* (5.7-fold)-; proline—*PUT1* (55.3-fold), *PUT4* (12.3-fold)*, PUT2* (6.0-fold), -and urea—*DUR3* (15.4-fold), *DUR1,2* (7.7-fold), as well as the regulator of nitrogen catabolite repression *DAL80* (11.9-fold). Altogether these observations reinforce the concept that in single-culture *S. cerevisiae* is sensing nitrogen limitation, this being confirmed, at least in part, by the lower levels of ammonium available registered at 48 h in the single-culture fermentation, compared to the levels registered in the mixed culture fermentation (Table 1). Notably, we found that the strain of *H. guilliermondii* used exhibits a particularly high proteolytic activity, which could contribute to enrich the medium in amino acids in the mixed-culture fermentation.

The most significantly overrepresented category among the genes that were found to be more expressed in the mixed-culture fermentation is “amino acid biosynthesis” (Table 2), which is consistent with the higher ammonia levels present in the growth medium [24]. In particular, several genes involved in biosynthesis of aromatic amino acids (five out of 12), serine (two out of 4), histidine (four out of 11), tryptophan (three out of five), lysine (two out of eight), serine (two out of four), threonine (three out of six), arginine (two out of ten) and lysine (two out of eight) were found to be up-regulated in response to the presence *of H. guilliermondii*. Interestingly, almost all the genes required for biosynthesis of the purine nucleotide monophosphate are more expressed in the mixed-culture fermentation: *ADE1* (4.8-fold), *ADE2* (4.3-fold), *ADE4* (4.2-fold), *ADE5,7* (5.2-fold), *ADE12* (3.8-fold) and *ADE17* (5.7-fold). It is possible that the higher expression of these genes in the mixed culture could result from the higher concentration of ammonium present in the growth medium since this has been found to exert a negative effect in the uptake of adenine [44]. Indeed, previous studies also report up-regulation of ADE genes during wine fermentations performed in the presence of ammonium [45].

Higher mRNA levels of several stationary growth-phase associated genes [46] were also obtained in mixed-culture at 48 h, namely *SNO1* (14.2-fold) and *SNZ3* (4.0-fold). Since at this point of the fermentation *S. cerevisiae* cells have ceased growth, both in the mixed-culture and in the single-culture fermentations (Fig. 1), the transcriptional activation of these genes is more likely to reflect the limitation of vitamins in the growth medium, as these genes were also found to be up-regulated under these conditions [47]. Also the increased expression of genes involved in biotin biosynthesis—*BIO3* (3.4-fold) and *BIO5* (3.3-fold)—and in the uptake of thiamine—*PHO3* (10.1-fold)—supports this hypothesis, as these genes’ expression has been described to be regulated in the dependence of the concentration of these vitamins present in the growth medium.

#### Fermentation stage 3 (96 h)

At the final fermentation stage analyzed, the expression of 214 *S. cerevisiae* genes was significantly altered in both fermentations, 71 genes being more expressed in mixed-culture and 143 in the single culture (Table 2) (Additional file 4). More than 38 % of the genes found to be more actively transcribed in the mixed culture (27 out of 71) have no known biological function. Among those that do have an associated biological function, we found *BIO3* (3.1-fold) and *BIO5* (2.5-fold) which are involved in biotin biosynthesis, reinforcing the suggestion that depletion of vitamins is one of the main consequences of mixed-culture fermentations. The dataset of genes found to be more expressed in the single culture at this fermentation stage was very broad in terms of physiological function, not being possible to identify significantly enriched functional classes.

### Analysis of the expression of genes related to aroma compounds production

**Fig. 5:**
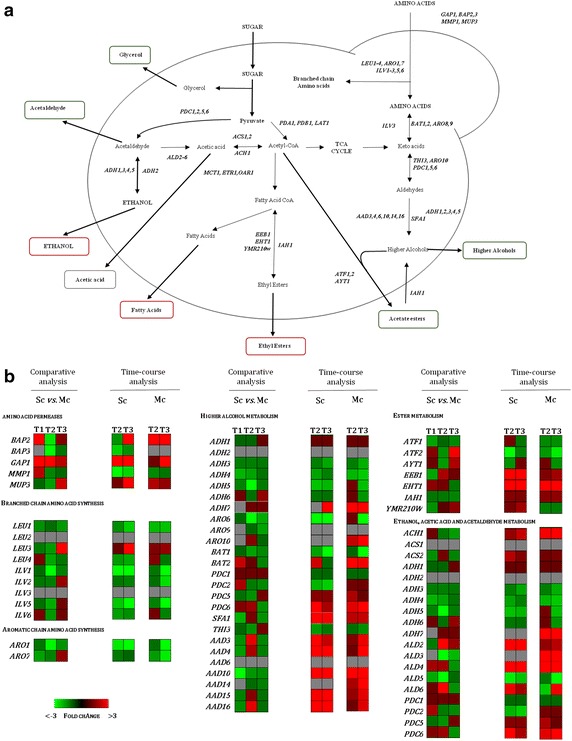
Biochemical pathways involved in flavor-active compounds formation. **a** Yeast genes encoding the enzymes that catalyze each step in the different pathways are shown in* italic*. **b** Expression of genes involved in aroma compounds formation: (1) comparison of Sc vs Mc gene expression at each fermentation stage, T1 (24 h), T2 (48 h) and T3 (96 h) *red* higher expressed in Sc and *green* higher expressed in Mc—Comparative analysis; and dynamics of genes expression along each fermentation. In this case ratios were obtained using the corresponding T1 as reference—Time-course analysis (*red* up-regulated and *green* down-regulated)

**Fig. 6:**
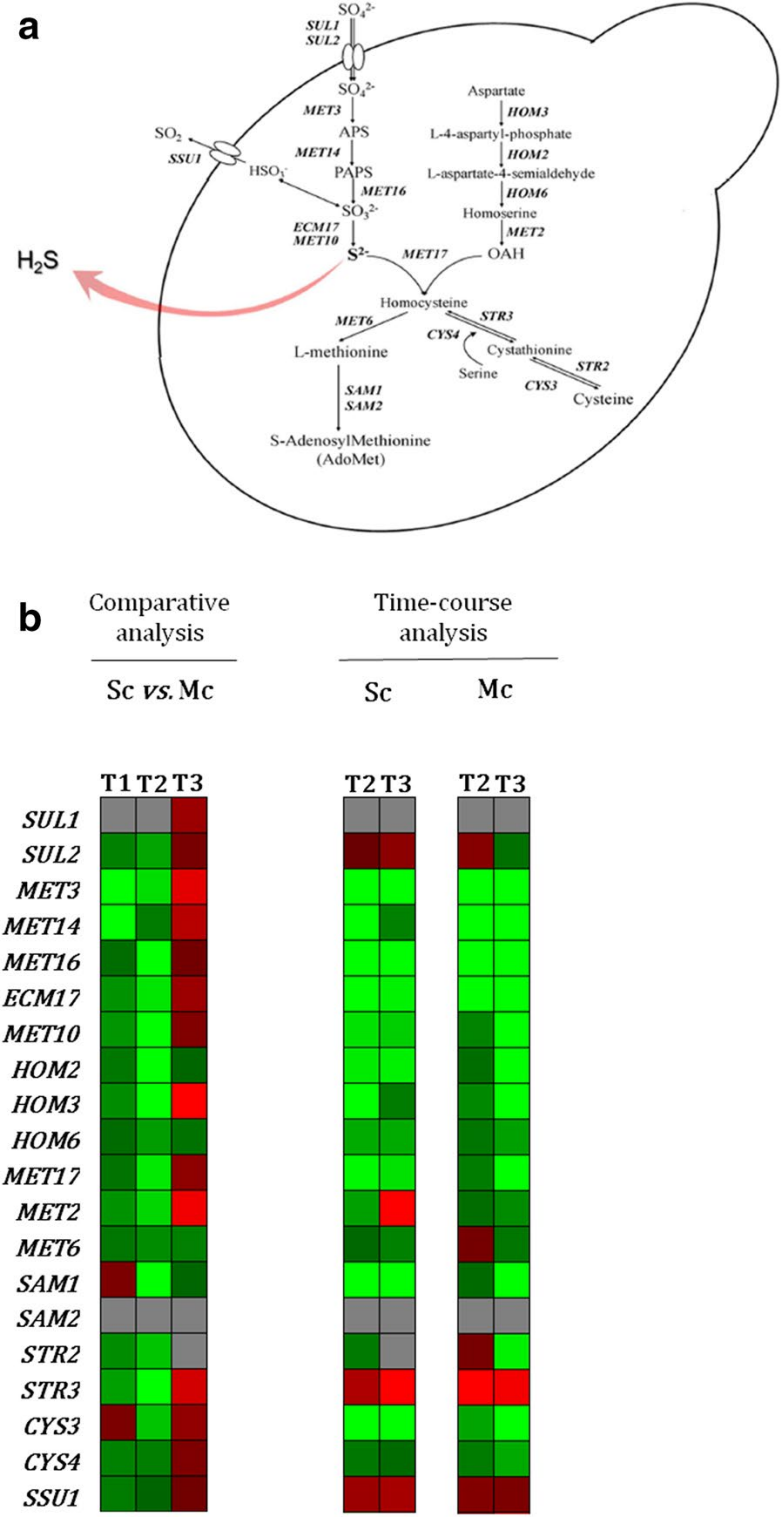
Biochemical pathways involved sulfur amino acid biosynthesis in *S. cerevisiae*. **a** Yeast genes encoding the enzymes that catalyze each step in the different pathways are shown in* italic*. **b** Expression of genes involved in hydrogen sulfide (H_2_S) formation: (1) comparison of Sc vs Mc gene expression at each fermentation stage, T1 (24 h), T2 (48 h) and T3 (96 h)—Comparative analysis (*red* higher expressed in Sc and *green* higher expressed in Mc) and dynamics of genes expression along each fermentation. In this case ratios were obtained using the corresponding T1 as reference—Time-course analysis (*red* up-regulated and *green* down-regulated)

The production of volatile compounds in the final wines was found to be significantly affected when *S. cerevisiae* was cultivated in the presence of *H. guilliermondii* [6]. While higher alcohols, acetate esters and acetaldehyde were highly detected in the wines fermented by mixed-culture of these two yeasts, the levels of ethyl esters, ethanol and H_2_S were more abundant in the wines that were only fermented by *S. cerevisiae* (Additional file 5). Transcriptome analysis of genes related to aroma production in *S*. *cerevisiae* have proven, at some extent, to be correlated with aroma compounds production during wine [22, 48, 49] and beer fermentation [50]. Given this, we have compared the expression of *S*. *cerevisiae* genes involved in the formation of different aroma compounds during single-fermentation or in the mixed fermentation with *H. guilliermondii* and the results obtained are summarized in Figs. 5 and 6. The variation of the expression of these genes along the two fermentations is also shown. The results show that, aside quantitative variation for each gene found within the different fermentations, most of them displayed the same trend in each fermentation. In the following sections are detailed the differences found in the expression of genes involved in production of higher alcohols, acetate and ethyl esters and H_2_S.

### Higher alcohols

Higher alcohols formation entails the activity of amino acid transporters, transaminases, decarboxylases and dehydrogenases. Amino acid permeases are encoded by *GAP1, BAP2, BAP3, MMP1* and *MUP3* genes [51], branched-chain amino acids transaminases by *BAT1* and *BAT2* genes, aromatic amino acids transaminases by *ARO8* and *ARO9* genes, decarboxylases encoded by *PDC1, PDC5*, *PDC6*, *THI3* and *ARO10,* and dehydrogenases by *ADH1*, *ADH2*, *ADH3*, *ADH4*, *ADH5, ADH6, ADH7* and *SFA1* [51, 52] (Fig. 5). Furthermore, aryl-alcohol dehydrogenases, *AAD10* and *AAD14,* are believed to be responsible for the degradation of aromatic aldehydes into their corresponding higher alcohols [53]. The increased levels of higher alcohols in mixed-culture fermented wines (Additional file 5) was in line with the higher expression of the majority of the genes involved in their metabolism that was observed under these conditions, comparing to the expression registered in the single-culture. The higher expression of *BAT1* and of genes involved in isoleucine-valine-leucine biosynthesis pathway (*LEU* genes and *ILV* genes) is also in agreement with the higher levels of isobutanol and 2-methyl-1-butanol detected in the wines produced by the mixed-cultures (Additional file 5). Despite the expression of *ARO1*, *ARO7*, and *ARO8* genes, involved in aromatic amino acid biosynthesis, was higher in the mixed-culture fermentation (Additional file 5), the amount of 2-phenylethanol produced was similar to the one produced by the single-culture (Additional file 5). These results are in agreement with those obtained by Rossouw et al. [22] who found a modest correlation between the expression levels of these three genes and 2-phenylethanol production.

### Acetate esters

The formation of acetate esters results from the condensation of acetyl-CoA with higher alcohols by acetyl transferases, encoded by the *ATF1* and *ATF2* genes [54–56]. *AYT1* gene, encoding a transferase of unknown substrate specificity, was also found to be associated with production of acetate esters production [32], while *IAH1*, encoding an esterase that preferentially acts on isomyl acetate, is associated to a decrease in acetate esters production [57]. Taking all this information in consideration, the higher levels of isoamyl-acetate, ethyl acetate and 2-phenyethyl acetate found in the mixed-culture fermented wine were positively correlated with higher expression levels of *ATF1* throughout the overall fermentation, and with higher expression of *ATF2* and *AYT1* at 48 h. Although *IAH1* was more actively transcribed in the mixed-culture this did not led to a reduced production of acetate esters, consistent with the results obtained by Molina et al. [48]. Taken together these results confirm the idea that acetate ester accumulation requires an appropriate control of these two opposed enzymatic activities in yeast [57].

### Ethyl esters

In ethyl ester formation, the condensation of acyl-CoA with ethanol is catalyzed by acyl-transferases, encoded by the *EEB1* and *EHT1* genes and *YMR210W* [58]. Nevertheless, similarly to acetate esters, ethyl esters might be degraded by the *IAH1*-encoded esterase [55]. Accordingly, the higher expression of *EEB1*, *EHT1* and *YMR210W* along with the lower expression of *IAH1* in *S. cerevisiae* in single-culture, could explain the higher levels of ethyl-esters detected in these wines (Additional file 5).

### Ethanol, acetaldehyde and acetic acid

The two final steps of alcoholic fermentation involves the decarboxylation of pyruvate, catalyzed by pyruvate decarboxylases (PDC), yielding acetaldehyde which in turn is reduced by the activity of several iso-enzymes of alcohol dehydrogenase (ADH) to ethanol. In *S. cerevisiae* there are three pyruvate decarboxylases, *PDC1*, *PDC5* and *PDC6* but only *PDC1* and *PDC5* are assumed to be active in yeast during fermentation [59]. Also five alcohol dehydrogenases are found in *S. cerevisiae, ADH1*-*5* which can in principle catalyze the reaction in both directions (i.e. acetaldehyde-to-ethanol and ethanol-to-acetaldehyde), although with different catalytic efficiencies [60]. The cytosolic *ADH1* gene product is the major enzyme responsible for converting acetaldehyde to ethanol [61]. Acetaldehyde can also be reduced to acetate by the action of aldehyde dehydrogenases encoded by *ALD2*-*6* [62, 63]. It has been hypothesized that *ALD4* and *ALD6* are the major contributors of acetate formation during wine fermentations [63], since *ALD3* and *ALD5* seem to be glucose-repressed [64].

Surprisingly, at the early stages of fermentation the mixed-culture produced higher levels of ethanol and at a faster rate, compared to the *S. cerevisiae* single-culture (Table 1). Hitherto, the ethanol levels present in the growth medium in the end of single-culture fermentation were significantly higher than those of the mixed culture wines (Additional file 6). Milanovic et al. [30] have also reported the same trend of ethanol production in mixed-culture fermentations of *S. cerevisiae* with *Starmerella bombicola*. The higher ethanol production at the earlier stages of mixed-culture fermentations is particularly intriguing since sugars consumption was higher in the single-culture fermentations. In this study, the higher expression of *ADH* genes in mixed-culture fermentation could be associated to such observation but do not explain the less ethanol obtained in the end of fermentation. One possible explanation could be the rerouting of the carbon flux towards glycerol leading to the decrease in ethanol yield, and increase in acetaldehyde levels. The significantly higher levels of glycerol and acetaldehyde obtained in wines fermented by *S*. *cerevisiae* and *H. guilliermondii* supports this assumption (Table 3; Additional file 6). Moreover the higher expression of *PDC1*, *PDC2* and *PDC5* and the reduced expression of *ALD2*, *ALD4* and *ALD6* in the mixed culture can also underlie the increased acetaldehyde concentration that was obtained in these wines. Surprisingly, this decreased expression of *ALD* genes in the mixed culture did not led to lower levels of acetic acid in the fermented wine (Additional file 6).

### H_2_S

**Fig. 7:**
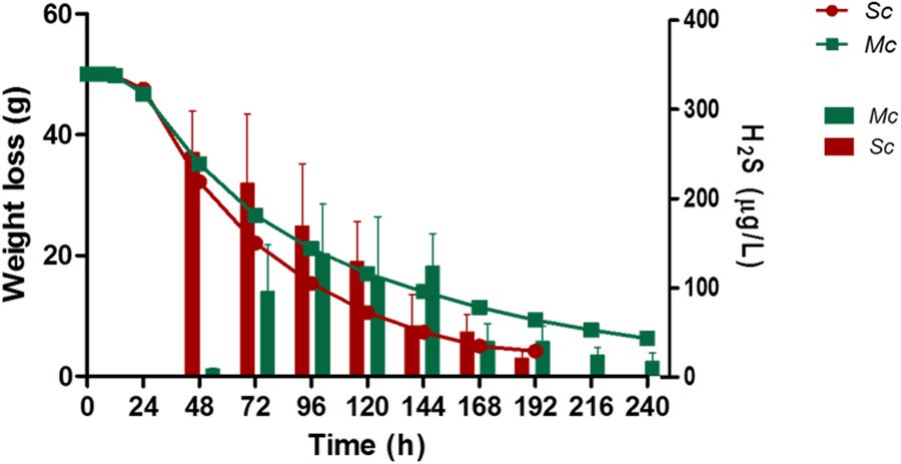
Hydrogen sulfide (H_2_S) liberation in single-culture—Sc (*red*) and mixed-culture—Mc (*green*) fermentations. Data points are the mean from triplicate fermentations ± SD

H_2_S production during wine fermentation results largely from the enzymatic activity of the Sulfate Reduction Sequence (SRS) pathway (Fig. 6a). The effect of *S*. *cerevisiae* cultivation in the presence of *H. guilliermondii* in the expression of genes involved in this pathway is shown in Fig. 6b. As seen for the other genes involved in the formation of other volatile compounds, aside quantitative variation for each gene found within the different fermentations, most of them displayed the same trend in each fermentation. It is known that *MET* genes expression is tightly correlated with yeast growth [65]. Indeed, most of the genes of the SRS pathway were highly expressed at the beginning of fermentation, where no H_2_S could be detected, being down-regulated in the later stages, coinciding with H_2_S liberation (Fig. 7). The higher expression of SRS genes in the mixed-culture fermentation does not correlate with the lower levels of H_2_S liberation observed. It is possible that this is the result of the higher expression of *MET10*, *MET5*, *MET17* and *MET2* genes, since their activity was correlated with reduced H_2_S production, [66–68]. On the overall it becomes evident that genes that impact H_2_S liberation during wine fermentation are under a tight regulatory control both during biosynthesis (*MET5* and *MET10*) and sulfide incorporation (*MET17*, *MET2*). Also, the results obtained in this study are not in agreement with the previous suggestion [69] that correlated high sulfide production with a higher expression of genes involved in the biosynthesis of thiamine.

## Conclusions

In this study, a transcriptomics-based approach was used to examine how *H. guilliermondii* impacted molecular responses of a *S. cerevisiae* wine yeast strain during a wine fermentation. This genome-wide analysis detected a large set of *S. cerevisiae* genes differentially expressed as a result of the presence of *H. guilliermondii* in the must. Several changes that could be detected in the transcriptome of *S. cerevisiae* appear to result from a cellular response to changes in nutrient availability in the fermenting must attributable to *H. guilliermondii* metabolic activity. These observations are of paramount interest since it is well recognized the effect of nitrogen availability on yeast growth and fermentation kinetics and on the production of the major metabolites arising from sugar fermentation that establish the wine aroma profile. Indeed, the presence of *H. guilliermondii* dramatically influenced the expression patterns of various flavor-active compounds associated genes, which could underlie the differences obtained on the aroma profiles of the wines. These findings raise the question whether the impact of non-*Saccharomyces* strains on the sensorial profile of wines results from an additive production of aroma compounds and/or from influencing the metabolic behavior of the fermentative yeast *S*. *cerevisiae* through modulation of the must nutritional properties.

In sum, our study underline the importance of such a global approach for the study of yeast–yeast interactions shedding light on the molecular basis of yeast dynamics during wine fermentation. This new information will be useful for the rational development of mixed-starter cultures o be use in winemaking industry.

## Methods

### Yeasts strains

A strain of *H. guilliermondii*, previously isolated in our laboratory from a fermenting grape-juice from Douro Region [70], was selected for this study based on interesting oenological traits such as high ethanol tolerance and low potential for hydrogen sulfide production. *S. cerevisiae* UCD522 was supplied by the Enology Culture Collection, Department of Viticulture and Enology, University of California, Davis, USA.

### Fermentation conditions and aroma compounds analysis

Fermentation conditions are described in Lage et al. [6]. Briefly, *S. cerevisiae* UCD522 and a natural *H. guilliermondii* strain were used to conduct alcoholic fermentation, of a natural grape-juice, either in single or mixed-culture. The initial pH of grape-juice was 3.26 and the concentration of sugars and nitrogen were 23.4 Brix and 387 mg YAN/L, respectively. Starter cultures of each strain were prepared by growing the yeast overnight in 100 mL-flasks, containing 50 mL of synthetic grape-juice medium with 267 mg YAN/L, supplied as DAP [19]. The flasks were incubated at 25 °C in an orbital shaker set at 150 rpm. Each yeast species was inoculated at a cell count of 10^6^ CFU/mL. The fermentations were conducted in 500 mL-flasks filled to 2/3 of their volume fitted with a side-arm port sealed with a rubber septum to allow anaerobic sampling, and were maintained at 20 °C in an orbital shaker set at 120 rpm. Samples were collected daily for assessing fermentation and growth parameters and, at the end of fermentations, for chemical analysis. Growth and fermentation parameters as well as the final concentration of aroma compounds in the wines can be found in Lage et al. [6].

### Analytical determinations

Glucose, fructose, glycerol and ethanol extracellular levels in the samples collected at the time points selected for transcriptomic analysis were determined with commercial biochemical kits (NZY Tech, Lda).

### Microarray and expression data analysis

Cell samples for DNA microarray analysis were obtained from both single- or mixed culture fermentations (Sc or Mc, respectively) at three different points: 24, 48 and 96 h after inoculation. Total RNA extraction was performed according to the hot phenol method. Concentration and purity was determined by spectrophotometry and integrity was confirmed using an Agilent 2100 Bioanalyzer with a RNA 6000 Nano Assay (Agilent Technologies, Palo Alto, CA, USA). RNA was processed for use on Affymetrix (Santa Clara, CA, USA) GeneChip Yeast Genome 2.0 Arrays, according to the manufacturer’s GeneChip 3′ IVT Express kit user manual. Briefly, 100 ng of total RNA containing spiked in Poly-A RNA controls was used in a reverse transcription reaction (GeneChip 3′ IVT Express Kit; Affymetrix) to generate first-strand cDNA. After second-strand synthesis, double-stranded cDNA was used in a 16 h in vitro transcription (IVT) reaction to generate aRNA (GeneChip 3′ IVT Express Kit; Affymetrix). Size distribution of the aRNA and fragmented aRNA, respectively, was assessed using an Agilent 2100 Bioanalyzer with a RNA 6000 Nano Assay. 5 µg of fragmented aRNA was used in a 100-µl hybridization cocktail containing added hybridization controls. 80 µl of mixture was hybridized on arrays for 16 h at 45 °C. Standard post hybridization wash and double-stain protocols (FS450_0003; GeneChip HWS kit, Affymetrix) were used on an Affymetrix GeneChip Fluidics Station 450. Arrays were scanned on an Affymetrix GeneChip scanner 3000 7G.

Scanned arrays were analyzed first with Affymetrix Expression Console software for quality control. Subsequent analysis was carried out with DNA-Chip Analyzer (dChip) 2010 (http://www.dchip.org, Wong Lab, Harvard) applying a probe set mask file excluding all probes on the array representing *Schizosaccharomyces pombe* transcripts. The arrays were normalized to a baseline array with median CEL intensity by applying an Invariant Set Normalization Method [71]. Normalized CEL intensities of the 16 arrays were used to obtain model-based gene expression indices based on a PM (Perfect Match)-only model [72]. Replicate data for the same sample type were weighted gene-wise by using inverse squared standard error as weights. All genes compared were considered to be differentially expressed if the 90 % lower confidence bound of the fold change between experiment and baseline was above 1.2. The lower confidence bound criterion means that we can be 90 % confident that the fold change is a value between the lower confidence bound and a variable upper confidence bound. Li and Hung Wong [72] have shown that the lower confidence bound is a conservative estimate of the fold change and therefore more reliable as a ranking statistic for changes in gene expression.

As *H. guilliermondii* RNA was present in mixed-culture RNA, there was a potential warning within this experiment related with the possible cross-hybridization of *H. guilliermondii* RNA on *S. cerevisiae* arrays which was considered. In this line, a total of 1.5 µg of *H. guilliermondii* genomic DNA was labelled using the Bioprime DNA labelling System (Invitrogen) following a strategy for genomic DNA hybridizations to GeneChips developed by Hammond et al. [73]. Cleanup was performed using MinElute PCR Purification kit (Qiagen) and quality was checked on an Agilent 2100 Bioanalyser using a DNA 1000 assay. Five micrograms was analyzed on Affymetrix GeneChip Yeast Genome 2.0 Arrays following the protocol described above for RNA samples. Genes whose signal was above the cut-off were designated cross-hybridizing genes, and were later removed from the analysis (223 ORFs).

### Statistical and bioinformatic analysis

To assess the changes in *S. cerevisiae* transcriptome in the response to the presence of *H. guilliermondii* throughout the fermentation, different analysis were performed. The transcriptomic data were first analyzed using a principal component analysis, PCA method. PCA was applied as an exploratory data analysis method to visualize differences between the diverse data sets. Also EPCLUST online software (http://ep.ebi.ac.uk/EP/EPCLUST/) was used for cluster analysis by the K-means method. For the identification of differentially expressed genes, the data were analyzed using Rank Product (RP) [74], as implemented in the MeV software [75]. RP, a nonparametric two-class unpaired method with a false discovery rate (FDR) multiple testing correction (*P* < 0.05, 5 % FDR) was used to identify differentially expressed genes between single- and mixed-culture fermentations, either irrespective of fermentation stage or at each fermentation stage separately. Comparisons by RP analysis were done using single-culture fermentations as one experimental group and mixed-culture fermentations as another experimental group, in order to identify specific genes associated with the response of *S. cerevisiae* to the presence of *H. guilliermondii*. The gene lists were analyzed for enrichment of functional categories using the FunSpec interpreter [76], available online at http://funspec.med.utoronto.ca.

### Microarray accession numbers

The microarrays hybridization data are available at the Gene Expression Omnibus (http://www.ncbi.nlm.nih.gov/geo) under accession number GSE66521.

### Validation of microarray data by qRT-PCR assays

To evaluate the overall quality of the microarray data we employed quantitative real-time (kinetic) PCR to amplify cDNA products reversely transcribed from mRNA (RT) (real time RT-PCR). We analyzed the expression of some genes, *THI20*, *DAL80*, *EEB1*, *ARO8*, *MEP2*, *BAT1*, *MET5* and *MET10*, using the same RNA from the original microarray experiments. In the Additional file 7 are depicted the sequences of the primers used in this analysis. Total RNA (5 µg) was reverse transcribed using SuperScript III Platinum (Invitrogen). 1 µL of the reverse-transcribed RNA was used as template to amplify the genes, using specific primers. Reaction mixtures contained 12.5 μL of SYBR Green supermix (Platinum SYBR Green qPCR Supermix UGD with Rox, Invitrogen), 0.8 μL each of the forward and reverse primers, 2 μL cDNA and sterile nuclease free H_2_O to a total volume of 25 μL.

Real-time qRT**-**PCR reaction conditions were 5 min at 95 °C for initial denaturation and activation of the DNA polymerase, followed by 50 cycles of denaturation at 95 °C for 30 s, annealing at the appropriate temperature specific for each primer pair for 30 s and extension at 60 °C for 30 s. The StepOne software (version 2.2.2, Applied Biosystems) was programmed to collect real time fluorescence data during the annealing and extension steps. Meltcurve analysis was performed after every qRT**-**PCR run to verify the specificity of the primers and to detect the presence (if any) of primer dimers. No-template controls were included for each primer pair. After RT**-**PCR, standard curves were plotted using the StepOne software (version 2.2.2, Applied Biosystems). The standard curve for each gene was generated from serial dilutions of cDNA [77]. The correlation coefficients and the amplification efficiency (E) of each reaction were then calculated from the slope of each trend line equation, according to the equation E = 10^(−1/slope)^. The expression of each gene determined by real-time qRT-PCR was normalized to the expression of the house-keeping gene, *ACT1* (encoding the structural protein actin). Relative quantification of the expression of each gene at each sampling point (24, 48 and 96 h) for *S. cerevisiae* in single- or in mixed-culture was determined by the ΔΔC_t_ method [78]. The qRT-PCR results correlated well with those obtained from the microarrays (Additional file 7). Additionally, qRT-PCR demonstrated that the expression measured in mixed-culture fermentations is specifically associated with *S. cerevisiae* by the lack of amplification observed in *H. guilliermondii* cDNA in single culture fermentation.

## Additional files

Additional file 1: Association between *S. cerevisiae* genes whose expression changed along the single or mixed wine fermentations with their documented regulators. Additional file 2: K-means clustering of differentially expressed genes during single-culture fermentations. Genes that showed at least two-fold altered expression in two or more consecutive time points during the single-culture fermentation were subjected to K-means clustering and grouped in ten clusters. Additional file 3: K-means clustering of differentially expressed genes during mixed-culture fermentations. Genes that showed at least two-fold altered expression in two or more consecutive time points during the mixed-culture fermentation were subjected to K-means clustering and grouped in ten clusters. Additional file 4: List of genes significantly affected (FDR<0.05) between *S. cerevisiae* in single- and mixed-culture. Additional file 5: Concentration of aroma compounds in the wines obtained with single-cultures of *Saccharomyces cerevisiae* UCD522 and *H. guilliermondii* or in consortium. Additional file 6: Overview of growth and fermentation parameters of wines obtained with single-cultures of *Saccharomyces cerevisiae* UCD522 and *H. guilliermondii* or in consortium. Additional file 7: Validation of microarray data using qRT-PCR.
