# Supplementary material for: Genomic expression program of Saccharomyces cerevisiae along a mixed-culture wine fermentation with Hanseniaspora guilliermondii
Source: Microb Cell Fact. 2015 Aug 28;14:124. doi: 10.1186/s12934-015-0318-1 (PMC4552253; doi:10.1186/s12934-015-0318-1)
Supplement: Additional file 1: — Association between S. cerevisiae genes whose expression changed along the single or mixed wine fermentations with their documented regulators. [file 12934_2015_318_MOESM1_ESM.pdf]

Additional file 3 - Association between *S. cerevisiae* genes whose expression was up-regulated along the single or mixed wine fermentations with their documented regulators

	Sc 24h	Sc 48h	Sc 96h	Mc 24h	Mc 48h	Mc 96h
Abf1p	292	161	234	225	80	135
Aca1p	0	1	0	0	0	0
Ace2p	20	17	14	14	6	8
Adr1p	58	78	110	41	31	84
Aft1p	55	27	39	37	11	19
Aft2p	8	18	37	6	5	13
Arg80p	15	6	10	15	5	2
Arg81p	11	2	13	12	2	4
Aro80p	10	13	20	6	7	8
Arr1p	2	5	3	3	3	3
Ash1p	18	17	24	10	7	16
Azf1p	11	2	9	6	1	5
Bas1p	32	16	17	19	14	5
Cad1p	43	68	28	32	39	34
Cat8p	4	5	10	3	0	8
Cbf1p	115	71	117	81	33	63
Cha4p	16	10	6	12	2	1
Crz1p	9	13	7	6	2	7
Cst6p	1	1	1	0	0	0
Cup9p	13	11	13	7	5	9
Dal81p	30	31	32	33	14	19
Dal82p	26	16	19	24	8	7
Ecm22p	19	5	23	10	1	7
Esa1p	2	0	0	2	0	0
Elp6p	0	0	1	0	0	1
Fhl1p	199	71	128	162	39	70
Fkh2p	70	34	35	52	16	18
Flo8p	43	29	50	31	12	35
Fzf1p	14	16	11	8	8	10
Gal4p	13	11	19	5	2	7
Gat1p	18	11	20	13	5	14
Gat3p	28	8	16	25	3	4
Gcn4p	80	56	51	62	53	29
Gcr1p	8	6	6	9	3	2
Gcr2p	18	9	13	15	4	6
Gln3p	21	20	21	25	7	12
Gsm1p	1	0	7	1	0	5
Gts1p	4	8	15	5	5	10
Haa1p	1	2	0	0	1	1
Hac1p	1	1	2	1	1	0
Hal9p	14	4	11	15	1	7
Hap1p	8	16	37	3	7	30
Hap2p	22	17	41	18	1	14
Hap3p	21	16	37	13	3	14

Hap4p	22	16	42	11	3	19
Hap5p	20	19	43	12	1	18
Hcm1p	32	33	49	29	15	26
Hmo1p	193	85	159	140	39	99
Hms1p	9	3	6	7	2	2
Hms2p	5	3	7	2	1	4
Hot1p	7	31	15	6	12	21
Hsf1p	23	33	49	18	11	30
Ifh1p	135	25	23	131	13	14
Ime1p	1	1	2	1	0	2
Ino2p	11	5	12	8	0	4
Ino4p	97	47	95	57	20	38
Leu3p	39	26	27	29	18	13
Mac1p	10	7	13	8	4	5
Mal13p	1	1	18	2	1	0
Mal33p	20	10	41	10	4	10
Mbp1p	68	22	52	53	13	18
Mcm1p	69	27	1	48	13	24
Met28p	14	5	7	11	4	2
Met31p	26	9	12	21	8	5
Met32p	56	28	82	44	18	51
Met4p	36	28	63	25	17	42
Mga1p	43	24	51	38	11	27
Mga2p	2	1	6	1	0	2
Mot2p	4	2	3	6	1	1
Mot3p	10	7	12	8	3	9
Msn1p	16	11	12	12	6	6
Msn2p	194	172	266	131	68	164
Msn4p	23	37	40	13	13	26
Mss11p	19	7	7	10	2	4
Ndd1p	7	0	1	5	0	0
Ndt80p	2	0	2	1	0	1
Oaf1p	54	40	78	41	17	43
Pdc2p	2	3	1	2	1	0
Pdr1p	50	45	61	44	19	40
Pdr3p	10	14	35	9	4	16
Pdr8p	1	0	0	0	0	0
Phd1p	80	49	94	60	23	62
Pho2p	24	21	11	22	11	9
Pho4p	38	30	47	24	18	20
Pip2p	21	14	42	21	3	17
Plm2p	23	16	27	23	9	10
Pog1p	11	4	9	14	1	2
Ppr1p	8	1	0	4	0	0
Put3p	15	18	24	11	4	16
Rap1p	303	139	207	230	63	120
Rds1p	3	4	5	1	0	2
Rds2p	20	24	70	12	8	48
Reb1p	77	39	47	62	15	25
Rgm1p	10	6	4	7	1	2

Rgt1p	5	4	9	7	1	3
Rlm1p	15	6	16	11	1	6
Rme1p	19	7	9	17	5	4
Rpn4p	15	17	31	9	2	12
Rsf2p	4	1	6	2	1	2
Rtg1p	10	6	12	3	2	4
Rtg3p	26	22	38	14	8	19
Rts2p	0	2	1	1	0	0
Sfl1p	4	2	7	3	1	1
Sfp1p	103	7	18	91	4	8
Sip4p	12	10	8	8	5	4
Skn7p	78	65	94	57	29	73
Sko1p	96	112	105	75	44	84
Smp1p	35	22	28	23	9	17
Sok2p	112	112	169	88	43	113
Spt23p	279	200	312	235	72	182
Srd1p	8	4	8	5	0	4
Stb1p	11	1	4	9	1	0
Stb5p	43	42	61	27	17	48
Ste12p	350	217	324	236	87	196
Stp1p	13	10	15	13	8	6
Stp2p	27	9	12	17	6	6
Sua7p	4	1	1	4	1	0
Swi4p	82	39	69	58	19	40
Swi5p	24	19	29	17	8	13
Swi6p	59	14	33	44	9	16
Tbf1p	83	45	69	54	20	44
Tec1p	73	61	100	66	25	69
Thi2p	7	5	9	4	2	3
Tos4p	22	15	23	23	6	12
Tos8p	43	28	45	35	8	17
Tye7p	22	7	13	17	6	7
Uga3p	10	6	9	10	2	4
Upc2p	5	1	7	2	1	3
Usv1p	5	4	3	3	2	3
War1p	4	2	2	5	1	1
Yap1p	80	95	99	66	48	64
Yap3p	3	1	4	5	1	1
Yap5p	89	85	78	80	46	64
Yap6p	161	127	144	109	62	116
Yap7p	16	26	32	14	15	17
YJL206C	3	4	7	2	2	5
Yrr1p	3	0	2	1	0	1
Zap1p	12	4	10	10	4	3

Additional file 3 - Association between *S. cerevisiae* genes whose expression were down-regulated along the single or mixed wine fermentations with their documented regulators

	Sc 24h	Sc 48h	Sc 96h	Mc 24h	Mc 48h	Mc 96h
Abf1p	331	334	253	225	146	153
Cbf1p	159	139	94	97	58	59
Cin5p	272	195	124	189	95	85
Dal80p	10	7	4	5	6	2
Dig1p	5	10	13	2	1	2
Elp6p	1	1	0	1	1	0
Fhl1p	161	214	172	123	66	144
Fkh1p	39	60	49	21	25	22
Fkh2p	57	77	59	29	27	37
Gal80p	1	0	0	1	1	0
Gat3p	21	33	26	10	5	23
Gsm1p	7	4	0	9	4	0
Gzf3p	9	4	3	5	1	2
Hir1p	9	9	11	4	5	5
Hir2p	14	10	4	6	8	3
Hmo1p	207	223	169	169	76	125
Hms1p	7	9	7	2	3	5
Hms2p	9	9	6	7	1	2
Ixr1p	27	23	18	21	13	15
Leu3p	40	46	34	27	15	25
Mcm1p	73	90	60	40	27	33
Mga1p	63	52	43	41	34	21
Mga2p	7	3	2	5	1	1
Mig1p	22	17	16	12	8	12
Mig2p	5	3	3	3	2	2
Mig3p	4	0	0	3	0	0
Mot3p	16	14	11	13	9	5
Nrg1p	77	43	25	55	30	9
Oaf3p	68	36	19	48	23	9
Plm2p	37	33	27	21	17	16
Pog1p	11	13	11	5	8	8
Rdr1p	1	1	0	1	1	0
Rds1p	8	3	4	3	0	1
Reb1p	70	78	81	41	36	37
Rfx1p	77	87	66	44	38	33
Rgt1p	12	10	6	5	4	6
Rim101p	21	22	13	15	10	5
Rme1p	11	20	20	6	3	16
Rox1p	36	34	26	29	20	14
Rph1p	19	16	11	6	3	7
Rsf2p	6	7	4	3	1	2
Rtg1p	16	15	11	6	0	3
Rtg3p	52	34	25	33	15	6
Rts2p	3	0	0	0	1	0

Sfl1p	8	5	4	4	1	2
Skn7p	134	104	68	107	55	46
Sko1p	177	113	69	117	60	51
Sok2p	237	151	100	178	90	64
Srd1p	12	10	9	5	4	4
Stb5p	88	53	35	68	36	22
Sua7p	1	4	2	0	1	3
Sum1p	28	21	13	22	9	6
Tbf1p	93	96	72	67	43	41
Tos4p	32	26	21	19	19	14
Tos8p	63	52	42	26	24	22
Tup1p	41	57	43	31	19	31
Ume6p	59	49	23	40	20	8
Usv1p	6	6	4	3	1	2
Xbp1p	79	69	48	53	34	30
Yap6p	227	193	133	169	78	92
Yhp1p	27	35	26	14	15	12
YJL206C	10	8	4	8	3	1
Yox1p	84	111	82	36	42	40
Yrm1p	344	381	336	193	150	195
