# Supplementary figures and images for: Genomic expression program of Saccharomyces cerevisiae along a mixed-culture wine fermentation with Hanseniaspora guilliermondii
Source: Microb Cell Fact. 2015 Aug 28;14:124. doi: 10.1186/s12934-015-0318-1 (PMC4552253; doi:10.1186/s12934-015-0318-1)

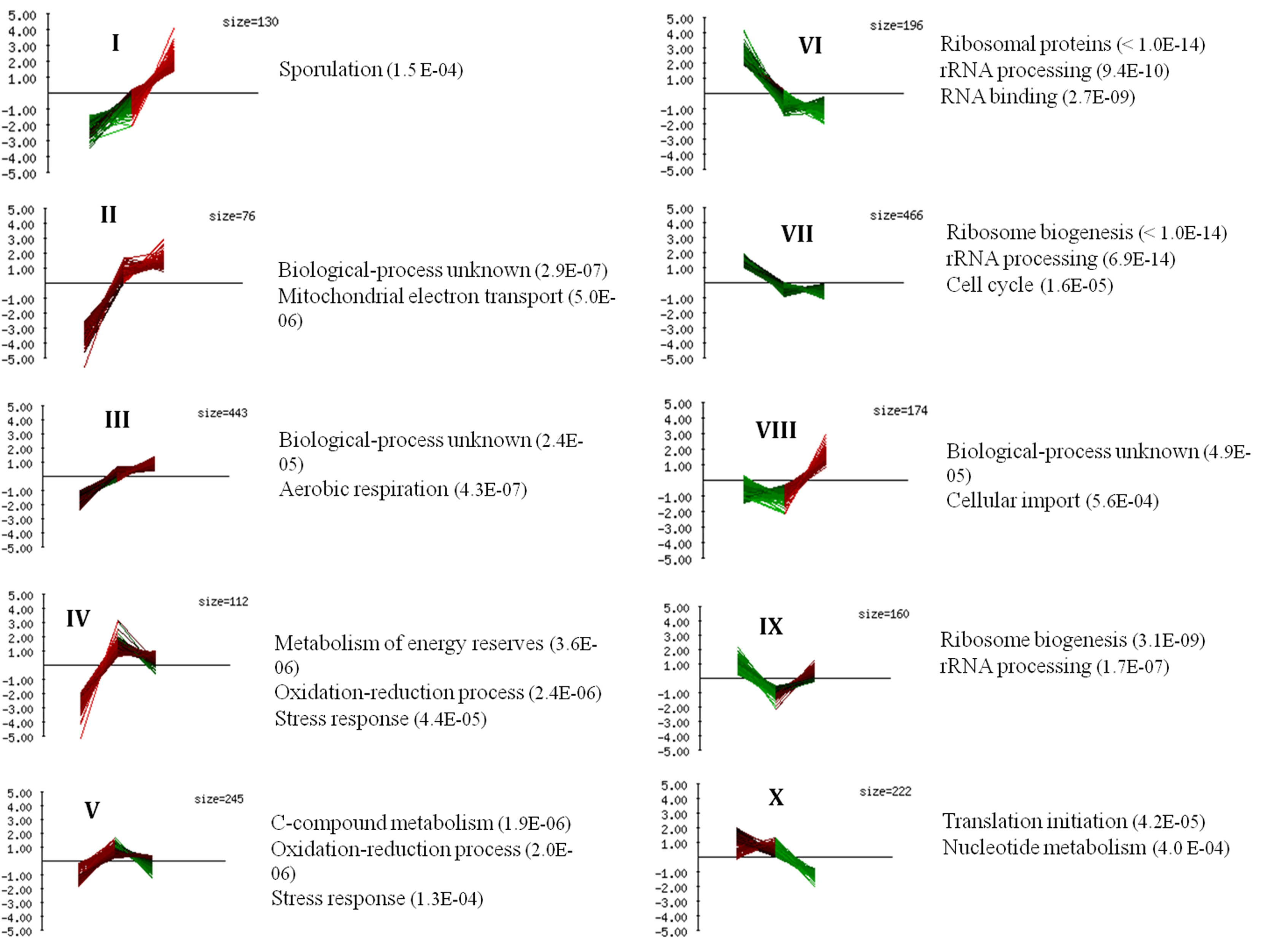

Supplement: Additional file 2: — K-means clustering of differentially expressed genes during single-culture fermentations. Genes that showed at least two-fold altered expression in two or more consecutive time points during the single-culture fermentation were subjected to K-means clustering and grouped in ten clusters. [file 12934_2015_318_MOESM2_ESM.tif]

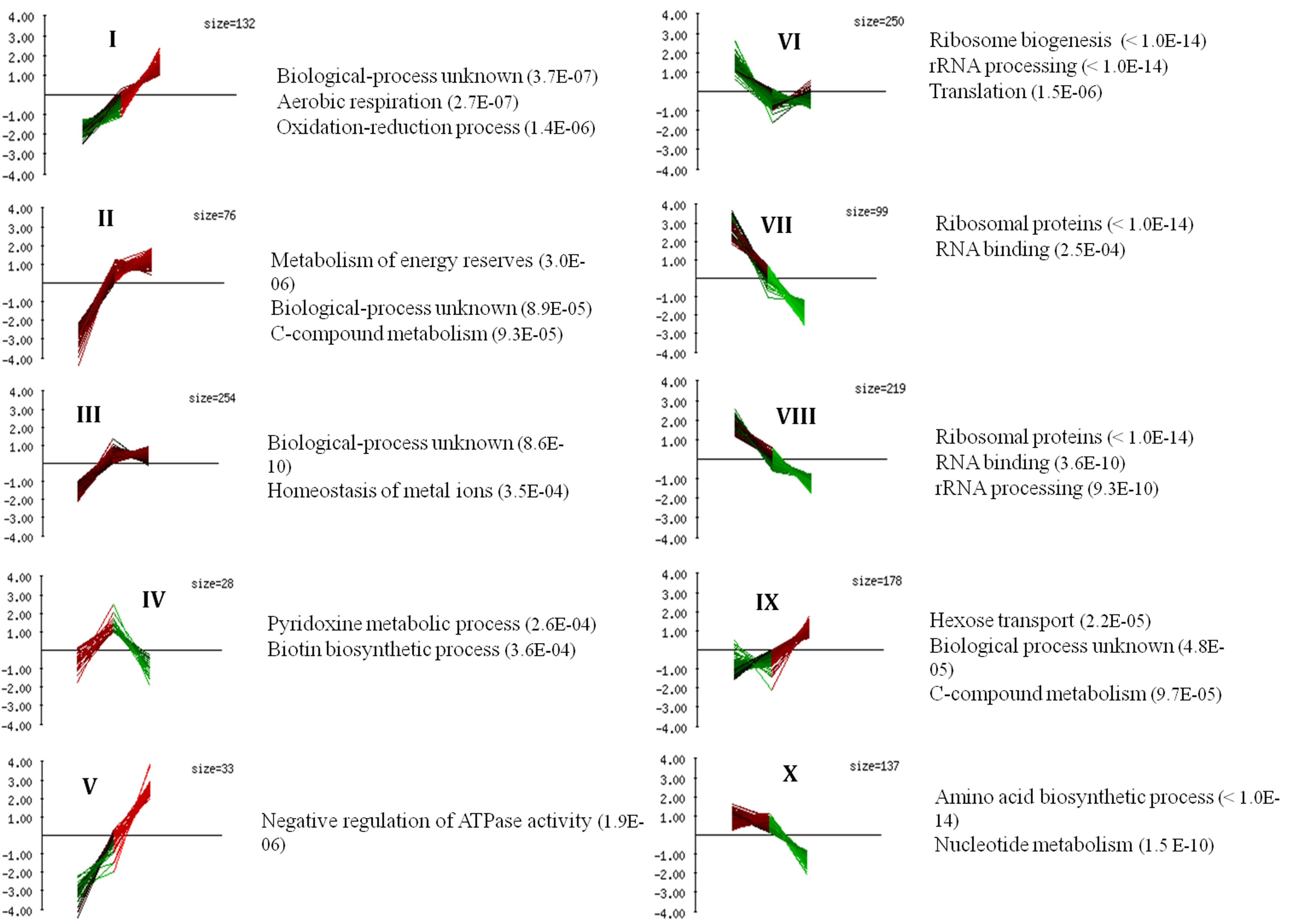

Supplement: Additional file 3: — K-means clustering of differentially expressed genes during mixed-culture fermentations. Genes that showed at least two-fold altered expression in two or more consecutive time points during the mixed-culture fermentation were subjected to K-means clustering and grouped in ten clusters. [file 12934_2015_318_MOESM3_ESM.tif]
