# Supplementary material for: Genomic expression program of Saccharomyces cerevisiae along a mixed-culture wine fermentation with Hanseniaspora guilliermondii
Source: Microb Cell Fact. 2015 Aug 28;14:124. doi: 10.1186/s12934-015-0318-1 (PMC4552253; doi:10.1186/s12934-015-0318-1)
Supplement: Additional file 5: — Concentration of aroma compounds in the wines obtained with single-cultures of Saccharomyces cerevisiae UCD522 and H. guilliermondii or in consortium. [file 12934_2015_318_MOESM5_ESM.pdf]

Additional file 5 - Concentration of aroma compounds in the wines obtained by single-cultures of *S. cerevisiae* UCD522 and *H. guilliermondii* or in consortium at 20°C, containing an initial nitrogen concentration of 387 mg/L. Data points are the means from triplicate fermentations. (Stem from Lage et al., 2014).

Compounds	OD	*S. cerevisiae*	Mixed-culture	*H. guilliermondii*	OT
**Ethyl Esters (mg/L)**					
Ethyl butyrate	apple	0.32 ± 0.04 [a]	0.31 ± 0.04 [a]	0.03 ± 0.00 [b]	0.02
Ethyl hexanoate	banana , green apple	0.57 ± 0.01 [a]	0.42 ± 0.06 [a,b]	0.02 ± 0.01 [c]	0.08
Ethyl octanoate	banana, pear, floral	0.94 ± 0.13 [a]	0.49 ± 0.15 [b]	0.00 ± 0.00 [c]	0.06
Ethyl decanoate	grape	0.76 ± 0.09 [a]	0.52 ± 0.23 [a]	0.03 ± 0.01 [b]	0.51
Ethyl dodecanoate		0.57 ± 0.06 [a]	0.34 ± 0.09 [b]	0.04 ± 0.01 [c]	0.64
Ethyl lactate	fruity , buttery	1.65 ± 0.32 [a,b]	2.76 ± 0.47 [a]	0.00 ± 0.00 [b]	150.00
	**TOTAL**	4.82	4.84	0.12	
**Acetate Esters (mg/L)**					
Hexyl acetate	apple, cherry, pear, floral	0.09 ± 0.01 [a,b]	0.10 ± 0.01 [a]	0.01 ± 0.00 [d]	0.07
Isoamyl acetate	banana , fruity	11.16 ± 0.97 [b]	14.50 ± 1.19 [a]	0.36 ± 0.09 [d]	0.16
Phenylethyl acetate	fruity	1.24 ± 0.03 [c]	3.73 ± 0.40 [b,c]	6.58 ± 1.14 [a]	1.80
Ethyl acetate	acetic acid	100.33 ± 16.72 [c]	275.98 ± 18.36 [a]	140.07 ± 7.56 [b,c]	12.00
	**TOTAL**	112.82	294.31	147.02	
**Acids (mg/L)**					
Isobutyric acid	rancid, butter, cheese	1.21 ± 0.03 [b]	2.60 ± 0.92 [b]	9.31 ± 0.24 [a]	2.30
Butyric acid	cheese , rancid	1.27 ± 0.03 [a]	0.00 ± 0.00 [b]	0.00 ± 0.00 [b]	0.17
Isovaleric acid	sweet , rancid	0.51 ± 0.01 [b]	0.41 ± 0.09 [b]	0.46 ± 0.04 [b]	0.03
Hexanoic acid	cheese , rancid	2.52 ± 0.09 [a]	1.52 ± 0.12 [b]	0.21 ± 0.03 [c]	3.00
Decanoic acid	fat , rancid	1.96 ± 0.07 [a]	1.56 ± 0.54 [a]	0.47 ± 0.19 [b]	6.00
Dodecanoic acid		0.14 ± 0.02 [a]	0.11 ± 0.06 [a]	0.42 ± 0.12 [a]	10.00
	**TOTAL**	7.62	6.19	10.87	
Acetaldehyde (mg/L)	sherry, nutty,bruised apple	24.25 ± 2.32 [b]	52.17 ± 8.49 [a]	8.58 ± 0.78 [c]	10000
**Alcohols(mg/L)**					
2-phenylethanol	rose, honey	25.86 ± 1.05 [b]	30.28 ± 12.01 [b]	28.73 ± 2.02 [b]	200.00
1-propanol	alcohol, ripe fruit	25.68 ± 1.23 [b,c]	42.23 ± 2.33 [a]	31.43 ± 1.31 [b]	306.00
Isobutanol	alcohol , nail polish	47.81 ± 2.98 [c]	118.67 ± 5.50 [a]	121.00 ± 7.00 [a]	75.00
2-methyl-1-butanol	alcohol , nail polish	58.75 ± 3.09 [b]	68.23 ± 5.71 [b]	54.43 ± 0.49 [b]	-
3-methyl-1-butanol	alcohol , nail polish	186.42 ± 11.42 [c]	185.44 ± 19.72 [c]	77.27 ± 0.78 [d]	30.00
Methionol	sweet , potato	647.67 ± 95.03 [a]	285.50 ± 95.03 [c]	659.00 ± 101.80 [a]	1.00
	**TOTAL**	344.52	444.85	312.86	
**Sulphur compounds (µg/L)**					
Methyl thioacetate	Cheese, vegetables	33.30 ± 2.78 [a]	22.60 ± 2.78 [a,b]	0.00 ± 0.00 [c]	-

2-Mercaptoethanol	Poultry, solvent	311.00 ± 77.95 [a,b]	171.00 ± 77.95 [b,c]	0.00 ± 0.00 [c]	450
2-Methyltetrahydrothiophen-3-one	Metallic, butane	90.30 ± 7.81 [a]	70.80 ± 7.81 [a]	69.30 ± 11.11 [a]	150
3-Mercapto-1-propanol	roasted, potato, broth	23.70 ± 4.05 [a,b]	35.20 ± 4.05 [a]	7.91 ± 2.17 [b]	60
3-(Ethylthio)-1-propanol	Rancid, sweaty	77.03 ± 10.03 [a,b]	58.17 ± 10.03 [b]	0.00 ± 0.00 [c]	-
4-(Methylthio)-1-butanol	Onion, garlic, earthy	208.67 ± 16.56 [a]	200.67 ± 16.56 [a]	173.33 ± 31.34 [a]	100
Dimethyl sulphone	Odourless	11.86 ± 3.79 [a]	16.57 ± 3.79 [a]	0.00 ± 0.00 [b]	-
Benzothiazole	Rubber	0.00 ± 0.000 [b]	14.65 ± 0.00 [a,b]	0.00 ± 0.00 [b]	50-200
3-Methylthiopropionic acid *	roasted, butter, rancid	0.00 ± 0.00 [b]	0.00 ± 0.00 [b]	0.00 ± 0.00 [b]	-
	**TOTAL**	1403.53	875.15	909.55	
**H_2S**	rotten eggs	926.12 ± 201.31 [a,b]	621.32 ± 201.31 [b,c]	178.84 ± 38.86 [c]	50-80

OD – odour descriptor; OT - odour threshold. Odor descriptors and odor threshold reported in the literature (Simpson, 1979; Guth, 1997, Francis and Newton, 2005; Peinado et al., 2004; Moreno et al., 2005; Swiegers et al., 2005; Moreira et al., 2010) expressed in the same units of the corresponding volatile compounds.

Values in the same line with different superscript letters are significantly different ($p < 0.05$)
