# Supplementary material for: Genomic expression program of Saccharomyces cerevisiae along a mixed-culture wine fermentation with Hanseniaspora guilliermondii
Source: Microb Cell Fact. 2015 Aug 28;14:124. doi: 10.1186/s12934-015-0318-1 (PMC4552253; doi:10.1186/s12934-015-0318-1)
Supplement: Additional file 6: — Overview of growth and fermentation parameters of wines obtained with single-cultures of Saccharomyces cerevisiae UCD522 and H. guilliermondii or in consortium. [file 12934_2015_318_MOESM6_ESM.pdf]

Additional file 6 - Overview of growth and fermentation parameters referent to the wines obtained by single-cultures of *Saccharomyces cerevisiae* UCD522 and *Hanseniapora guilliermondii* or in consortium at 20°C, containing an initial nitrogen concentration of 387 mg/L. Data points are the means from triplicate fermentations. (Stem from Lage et al., 2014).

Yeasts	Time to completion fermentation (h)	Final sugars(g/L)	Maximum fermentation rate (CO_2/h)	pH	Volatile acidity (g/L)	Ethanol (% v/v)	Final YAN (mg/L)
S. cerevisiae	168	0.13 ± 0.26^{b}	0.55 ± 0.02^{a}	3.03 ± 0.11^{a}	0.17 ± 0.02^{b}	14.2 ± 0.00^{a}	$37.33 \pm 2.02^{b,c}$
H. guilliermondii	-	133.9 ± 8.85^{a}	0.15 ± 0.01^{c}	3.21 ± 0.13^{a}	0.55 ± 0.06^{a}	7.2 ± 0.00^{c}	310.33 ± 29.14^{a}
Mixed-culture	240	2.73 ± 1.28^{b}	0.36 ± 0.01^{b}	2.99 ± 0.13^{a}	0.30 ± 0.16^{b}	13.6 ± 0.00^{b}	$46.67 \pm 8.08^{b,c}$

DAP – diammonium phosphate; YAN - yeast assimilable nitrogen

Values in the same column with different superscript letters are significantly different ($p < 0.05$)
