# Supplementary material for: Genomic expression program of Saccharomyces cerevisiae along a mixed-culture wine fermentation with Hanseniaspora guilliermondii
Source: Microb Cell Fact. 2015 Aug 28;14:124. doi: 10.1186/s12934-015-0318-1 (PMC4552253; doi:10.1186/s12934-015-0318-1)
Supplement: Additional file 7: — Validation of microarray data using qRT-PCR. [file 12934_2015_318_MOESM7_ESM.pdf]

Additional file 7 –

Table 1 Genes and primers sequences used in real-time qRT-PCR.

Gene	Forward and reverse sequences
MET5	TGGATGGTGACAACATTCGT / TGCTCAGCGACTTCTAATGG
MET10	AGAGGATTTGGTTACTCC / AGTTCCTCAAGAGATGGG
ARO8	CGACTTCCTAATTGTGGAAGAT / GCTTTGGAGAACTTTGTGC
THI20	TGTCGCTACTTCTGGTTCTTC / CTTTCTTTCCTCACCTAACAATTTG
DAL80	AAAAAGCTGAATAACAACAATGTGAA / GGAACGGTTTCCTTTGGTTTTAA
MEP2	ACGTTTTAGGAGCCCCATCT / AGGAAACAACCTTGCCCTCT
EEB1	TCGTACACACTTGGGACAAGTTG / CAGTCCTTGTTAGAAATTGTGTTAAAGTTC
BAT1	CCAAATCCATCCAAGCCAAG / AGCAGATGGGTCAAGAGAAAG
ACT1	GATTCTGAGGTTGCTGCTTTGG / GACCCATACCGACCATGATACC

Table2 Comparison of qRT-PCR and microarray transcript fold ratios for *S. cerevisiae* single versus mixed culture fermentations. Fold changes shown were calculated at each time point indicated.

	T1 (24h)		T2 (48h)	
	qRT-PCR fold ratio	microarray fold ratio	qRT-PCR fold ratio	microarray fold ratio
MET5	-7.74	-1.56	-3.99	-2.66
MET10	-6.14	-1.58	-2.10	-2.99
ARO8	-1.69	-1.07	-6.10	-5.69
THI20	-5.11	-5.60	-1.54	-1.33
BAT1			-4.23	-1.76
EEB1			10.69	1.84
MEP2			29.3	2.36
DAL80			21.20	11.95
